# Myricanol prevents aging‐related sarcopenia by rescuing mitochondrial dysfunction via targeting peroxiredoxin 5

**DOI:** 10.1002/mco2.566

**Published:** 2024-06-12

**Authors:** Shengnan Shen, Qiwen Liao, Peng Lyu, Jigang Wang, Ligen Lin

**Affiliations:** ^1^ State Key Laboratory for Quality Ensurance and Sustainable Use of Dao‐di Herbs, Artemisinin Research Center, and Institute of Chinese Materia Medica China Academy of Chinese Medical Sciences Beijing China; ^2^ State Key Laboratory of Quality Research in Chinese Medicine, Institute of Chinese Medical Sciences University of Macau Macau China; ^3^ Kobilka Institute of Innovative Drug Discovery, School of Medicine The Chinese University of Hong Kong Shenzhen Guangdong China; ^4^ Shenzhen Institute of Respiratory Disease Shenzhen People's Hospital (First Affiliated Hospital of South University of Science and Technology of China and Second Affiliated Hospital of Jinan University, China) Beijing China; ^5^ Department of Oncology The Affiliated Hospital of Southwest Medical University Luzhou China; ^6^ Department of Pharmaceutical Sciences and Technology, Faculty of Health Sciences University of Macau Macau China

**Keywords:** healthy aging, mitochondria, myricanol, peroxiredoxin 5, redox homeostasis, sarcopenia

## Abstract

Aging is a process that represents the accumulation of changes in organism overtime. In biological level, accumulations of molecular and cellular damage in aging lead to an increasing risk of diseases like sarcopenia. Sarcopenia reduces mobility, leads to fall‐related injuries, and diminishes life quality. Thus, it is meaningful to find out novel therapeutic strategies for sarcopenia intervention that may help the elderly maintain their functional ability. Oxidative damage‐induced dysfunctional mitochondria are considered as a culprit of muscle wasting during aging. Herein, we aimed to demonstrate whether myricanol (MY) protects aged mice against muscle wasting through alleviating oxidative damage in mitochondria and identify the direct protein target and its underlying mechanism. We discovered that MY protects aged mice against the loss of muscle mass and strength through scavenging reactive oxygen species accumulation to rebuild the redox homeostasis. Taking advantage of biophysical assays, peroxiredoxin 5 was discovered and validated as the direct target of MY. Through activating peroxiredoxin 5, MY reduced reactive oxygen species accumulation and damaged mitochondrial DNA in C2C12 myotubes. Our findings provide an insight for therapy against sarcopenia through alleviating oxidative damage‐induced dysfunctional mitochondria by targeting peroxiredoxin 5, which may contribute an insight for healthy aging.

## Graphical abstract

MY was found to protect aged mice against the loss of muscle mass and strength through scavenging ROS accumulation to rebuild the redox homeostasis. Combining biophysical and pharmacological profiles, peroxiredoxin 5 was discovered and validated as the direct target of MY. Furthermore, mechanistic studies showed that MY reduced reactive oxygen species accumulation and damaged mitochondrial DNA in myotubes through activating peroxiredoxin 5.

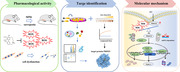

## 1 INTRODUCTION

Sarcopenia is the age‐related progressive loss of skeletal muscle mass, strength, and function, which is a generalized disorder seriously affects the elderly.^1^ Sarcopenia is more prevalent in older populations, and the muscle mass decline begins from approximately 40 years onward. The progression of sarcopenia can be accelerated by many conditions such as sedentary life style, unhealthy diet, and reduced physical performance.^2^ The mechanisms of sarcopenia are not clearly defined; inflammation, intramyocellular lipid deposition, insulin resistance, oxidative stress, and mitochondrial dysfunction have been considered to be correlated with sarcopenia.^3^

Accumulation of dysfunctional mitochondria, attributed by disruption of mitochondrial biogenesis, proteostasis, and dynamics, during aging, contributes to the complex etiology of sarcopenia.^4^ In skeletal muscle, mitochondria are the main source of reactive oxygen species (ROS). The homeostasis of ROS is important in maintaining mitochondrial integrity.^5^ An impaired redox homeostasis has been implicated in during aging. Skeletal muscle of aged organisms accumulates oxidative damage contributed by lipids, DNA, and proteins, leading to skeletal muscle dysfunction.^6^ Therefore, strategies that improving oxidative stress‐induced mitochondrial dysfunction could mitigate sarcopenia.^7^

Mammalian cells express three types of hydrogen peroxide eliminating enzymes including catalase, glutathione peroxidases, and peroxiredoxins (PRDXs). PRDXs are highly conserved by containing the cysteine‐dependent peroxidases, which effectively scavenge hydrogen peroxide and organic peroxides, significant in maintaining redox balance.^8^, ^9^ In 2‐Cys PRDXs, oxidation of the Cys residue to sulfonic acid leads to the inactivation of the peroxidase activity.^10^ PRDX5 is a member of PRDXs family, which has substrate specificity.^11^ The atypical PRDX5 is activated by forming an intramolecular disulfide bond with the internal resolving Cys instead of intermolecular disulfide bond as the typical 2‐Cys PPRDXs.^12^ The canonical PRDX5 is widely distributed in mitochondria while the noncanonical PRDX5 mainly locate in cytoplasm and peroxisome.^13^, ^14^ Both types of PRDX5 contain a conserved active motif PxxxTxxC that is critical for the peroxidase activity.^12^, ^15^ The decrease of PRDX5 causes excessive ROS production, leading to mitochondrial dysfunction.^16^ PRDX5 overexpression reduces mitochondrial ROS (mtROS) production, and suppresses lipid accumulation through AMP‐activated protein kinase pathway.^17^

We previously reported that myricanol (MY), a cyclic diarylheptanoid derived from Chinese bayberry, displays protective effects against insulin resistance in high‐fat diet‐fed obese mice and skeletal muscle wasting in dexamethasone‐stimulated mice.^18^, ^19^ The role of MY in mitigating sarcopenia and the molecular mechanism are yet to be elucidated. Herein, we provide evidence that MY could be a therapeutic agent to alleviate age‐associated skeletal muscle wasting and PRDX5 could be a potential target for mitigating sarcopenia.

## 2 RESULTS

### 2.1 MY mitigates the loss of muscle mass and strength in aged mice

**FIGURE 1:**
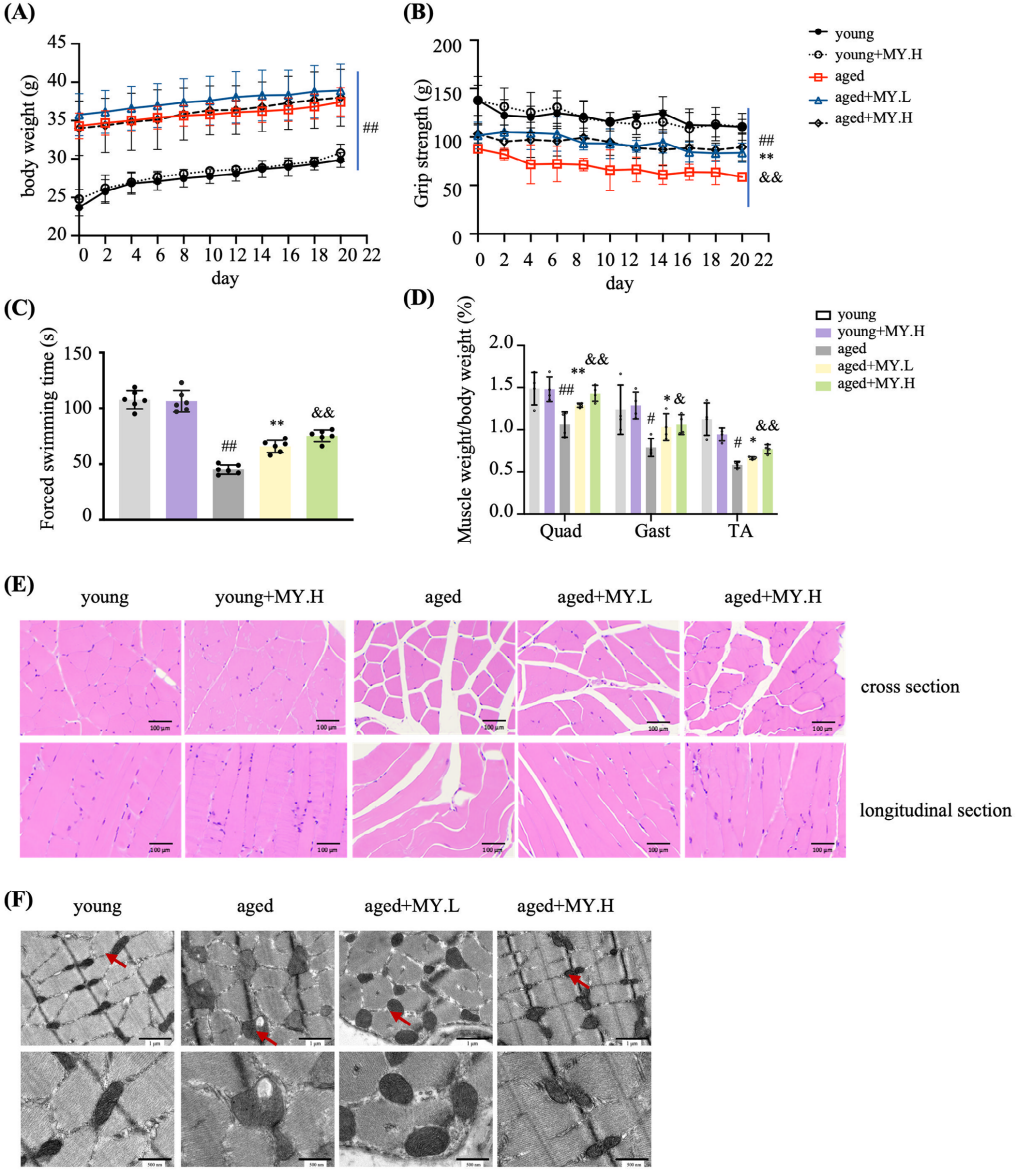
MY protects against aging‐related loss of muscle mass and strength in mice. (A) body weight. (B) Grip strength. (C) Forced swimming time. (D) The weight ratios of Quad, Gast, and TA muscle to the body weight. (E) Representative H&E staining of Gast. Top: cross section; bottom: longitudinal section. Scale bar = 100 µm. A microscope with 20× objective was used to capture the images. (F) Electron microscope analyses in TA muscle. Scale bar = 1 µm (top); Scale bar = 500 nm (bottom). Young: 3‐month‐old mice, PEG 400 solution; young+MY.H: 3‐month‐old mice, PEG 400 buffer with 50 mg/kg MY; aged: 18‐month‐old mice, PEG 400 solution; aged+MY.L: 18‐month‐old mice, PEG 400 buffer with 10 mg/kg MY; aged+MY.H: PEG 400 solution with 50 mg/kg MY. Data are displayed as mean ± SD, *n* = 6. ^#^ *p *< 0.05, ^##^ *p *< 0.01, young versus aged. *p < 0.05, **p < 0.01, aged versus aged+MY.L. ^&^ *p* < 0.05, ^&&^ *p* < 0.01, aged versus aged+MY.H.

To decipher the beneficial effect of the compound against age‐related muscle loss, 18‐month‐old male mice were recruited as aged group, and 3‐month‐old male mice were used as the young control. The body weight of mice increased with age and showed no difference after MY treatment in either young or aged mice (Figure 1A). The grip strength test revealed that MY treatment significantly enhanced muscle strength in aged mice, but did not affect the muscle strength of the young mice (Figure 1B). Furthermore, the forced swimming test showed that aged mice were prone to be exhausted when compared with young mice. The swimming exhaustive time of aged mice was shown to be longer after MY treatment (Figure 1C). Aging markedly reduced muscle index, but did not change liver index, whereas MY treatment greatly increased the muscle index of aged mice, especially for quadriceps (Quad), gastrocnemius (Gast), and tibialis anterior (TA), either low or high dosage (Figures 1D and S1). Therefore, MY improves muscle strength and prevents muscle loss in aged mice.

**FIGURE 2:**
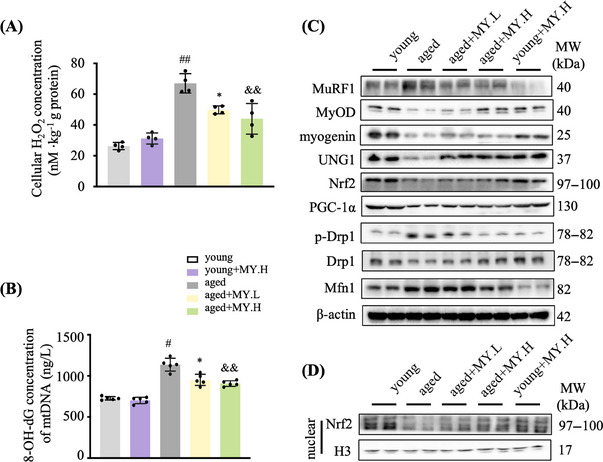
MY alleviates oxidative damage and mitochondrial dysfunction in skeletal muscle from aged mice. (A) Cellular H_2_O_2_ content. (B) 8‐OH‐dG level in mtDNA was measured by ELISA kit. (C) The expression of key proteins in muscle atrophy including MuRF1, myogenic differentiation including MyoD and myogenin, antioxidative capacity including UNG1 and Nrf2, and mitochondrial homeostasis including PGC‐1α, Drp1and Mfn1 in Gast muscle. β‐Actin was used as a loading control. (D) The expression of nuclear Nrf2. Histone H3 was used as the loading control. Young: 3‐month‐old mice, PEG 400 buffer; young+MY.H: 3‐month‐old mice, PEG 400 solution with 50 mg/kg MY; aged: 18‐month‐old mice, PEG 400 buffer; aged+ MY.L: 18‐month‐old mice, PEG 400 buffer with 10 mg/kg MY; aged + MY.H: PEG 400 buffer with 50 mg/kg MY. Data are shown as mean ± SD, *n* = 6. ^#^ *p *< 0.05, ^##^ *p *< 0.01, young versus aged. *p < 0.05, **p < 0.01, aged versus aged+MY.L. ^&^ *p* < 0.05, ^&&^ *p* < 0.01, aged versus aged+MY.H.

The skeletal muscle from young mice had normal architecture with dense appearance fibers, which possess similar size and are gathered in bundles uniformly and unfragmentally; whereas in aged mice, the muscle fibers showed degenerated appearance (Figure 1E). In aged mice, MY treatment maintained normal muscle architecture of young mice (Figure 1E). The diameter of muscle fiber in cross‐section showed no difference (Figure S2). Mitochondrial content and morphology vary greatly in skeletal muscle during aging.^20^, ^21^ Electron microscope (EM) images of TA indicated that the number of intramyofibrillar mitochondria was less in aged mice compared with young group (Figure 1F). Most of the mitochondria showed normal ultrastructure in the young group, possessing clear cristae and intact membrane in a mutually parallel manner; while in the aged mice, abnormal mitochondria were found, showing larger size, cristae loss, disrupted membrane, vacuolization, and matrix dissolution (Figure 1F). MY treatment obviously recovered healthy mitochondrial morphology in aged mice (Figure 1F). To analyze the oxidative products of mtDNA, 8‐hydroxydeoxyguanosine (8‐OH‐dG) in mitochondria from Gast was determined. The cellular H_2_O_2_ concentration from aged mice was significantly increased compared with the young group, and MY treatment dose‐dependently decreased the level of cellular H_2_O_2_ in Gsat from aged mice, but not in the young mice (Figure 2A). The level of 8‐OH‐dG in mitochondria from aged mice was significantly increased compared with the young group (Figure 2B). As expected, MY treatment dose dependently decreased the level of 8‐OH‐dG in Gsat from aged mice, but not in the young mice (Figure 2B). The Western blotting results showed that MY treatment decreased the expression of MuRF1 (muscle‐specific RING finger protein 1), an E3 ubiquitin ligase, and enhanced the expression of MyOD and myogenin, the markers of myogenic differentiation (Figure 2C). MY treatment upregulated the UNG1 and Nrf2 expression level in muscle from aged mice, to enhance the antioxidant and ROS scavenging capacity (Figure 2C). Furthermore, MY treatment induced Nrf2 protein accumulated and translocated to the nucleus, confirming that MY activates Nrf2 (Figure 2D). In addition, MY increased the expression level of PGC‐1α, which is a master regulator of mitochondria biogenesis (Figure 2C). A significant downregulation of Drp1 (dynamin‐related protein 1) expression and an upregulation of Mfn1 (mitofusion 1) were found in muscle from aged mice compared with the young mice (Figure 2C), which indicated aging shifts more mitochondria toward fusion. MY treatment reversed the above changes to rebalance the mitochondrial dynamics (Figure 2C). Overall, MY protects against aging‐associated loss of muscle mass and strength through mitigating oxidative damage‐induced mitochondrial dysfunction.

### 2.2 MY protects C2C12 myotubes against *tert*‐butyl hydroperoxide‐induced oxidative damage

**FIGURE 3:**
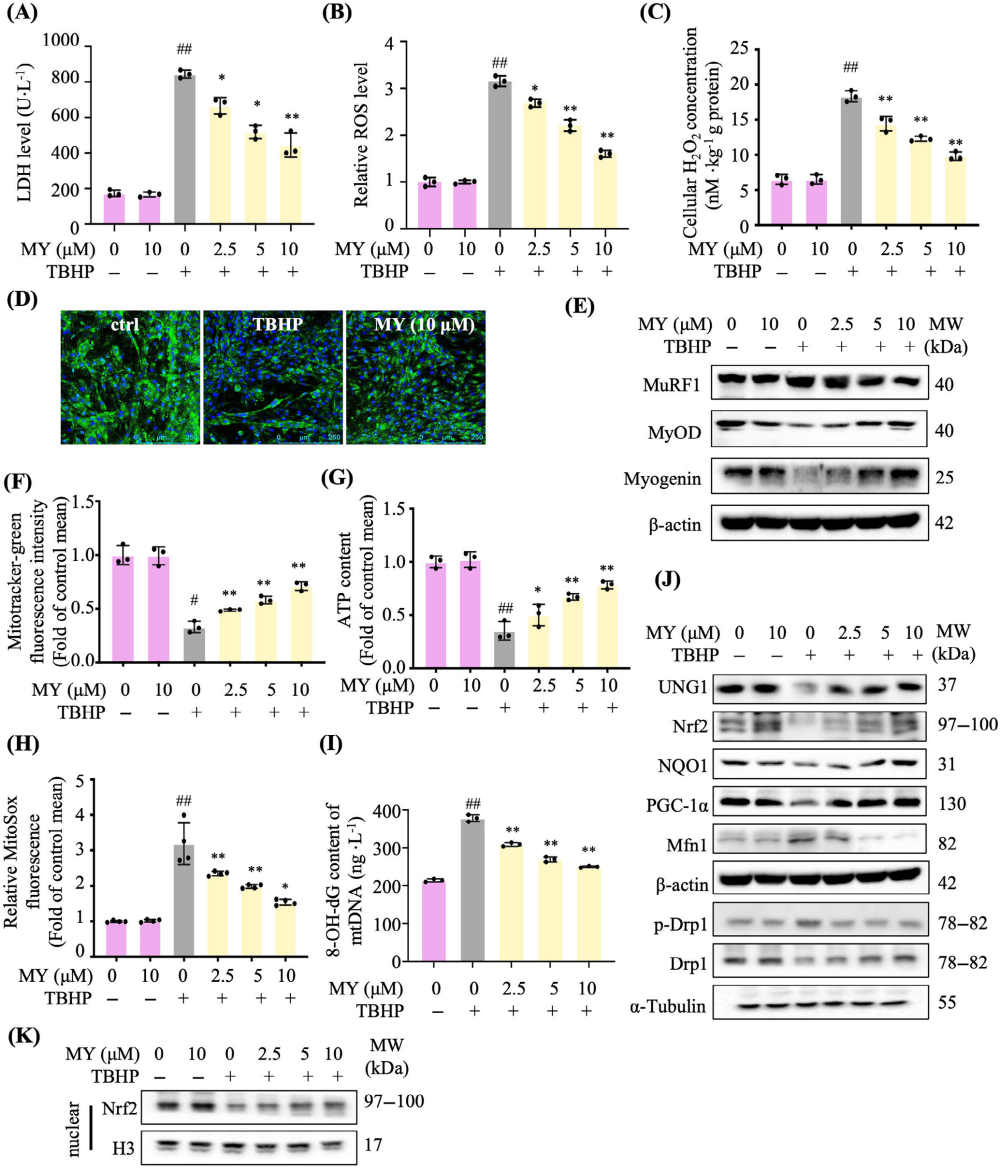
MY rescues C2C12 myotubes against TBHP‐induced oxidative damage through improving mitochondrial biogenesis and function. (A) LDH level in TBHP‐treated C2C12 myotubes. (B) ROS production. (C) Cellular H_2_O_2_ content. (D) Immunostaining of MyHC in C2C12 myotubes. (E) The expression levels of MuRF1, MyoD, and myogenin, β‐actin was used as control. (F) Mitochondrial content assessed by MitoTracker Green staining. (G) ATP production. (H) Mitochondrial ROS content determined by MitoSOX Red. (I) Cellular levels of 8‐OH‐dG in mtDNA from TBHP‐treated C2C12 myotubes was measured by ELISA. (J) The expression of key proteins in antioxidative capacity including UNG1, Nrf2 and NQO1, and mitochondrial homeostasis including PGC‐1α, Drp1 and Mfn1 in TBHP‐treated C2C12 myotubes. β‐actin was used as a loading control. (K) The expression of nuclear Nrf‐2. Histone H3 was used as a control. Data are displayed as mean ± SD, *n* = 6. #*p* < 0.05, ##*p* < 0.01, vehicle versus TBHP, *p < 0.05, **p < 0.01, MY versus TBHP.

In C2C12 myotubes, 200 µM *tert*‐butyl hydroperoxide (TBHP) was found to enhance ROS production, but did not cause significant cell death, which was used in the following experiments (Figure S3). As shown in Figure 3A, MY attenuated TBHP‐induced increase of LDH level in a dose‐dependent manner. MY was found to attenuate TBHP‐induced ROS production and cellular H_2_O_2_ accumulation dose dependently (Figures 3B and C). The content of MyHC (myosin heavy chain) was increased in MY‐treated cells assessed by immunostaining images (Figure 3D). Moreover, MY treatment decreased MuRF1 expression and increased MyOD and myogenin expression in TBHP‐treated C2C12 cells (Figure 3E). The above results indicated that MY attenuates TBHP‐induced oxidative damage in C2C12 myotubes.

### 2.3 MY improves mitochondrial biogenesis and function in TBHP‐treated C2C12 myotubes

As expected, TBHP stimulation obviously reduced the quantities of mitochondria, assessed by Mitotracker‐green staining and ATP content, whereas MY reversed the above changes dose dependently (Figures 3F and G). To determine whether MY enhanced antioxidative capacity to protect mitochondria, the mtROS was detected. MY treatment reduced TBHP‐induced increase of mtROS level in a dose‐dependent manner (Figure 3H). Consistently, MY treatment decreased the level of 8‐OH‐dG in TBHP‐treated C2C12 cells (Figure 3I). MY increased the protein expression levels UNG1, Nrf2, NQO1, and PGC‐1α (Figure 3J), and MY treatment induced Nrf2 protein accumulated and translocated to the nucleus (Figure 3K), indicating MY enhanced mitochondrial biogenesis and antioxidative capacity in C2C12 myotubes. And MY rebalanced the mitochondrial dynamics destroyed by oxidative damage via elevating the Drp1 expression and decreasing the Mfn1 expression (Figure 3J). Taken together, MY improves mitochondrial biogenesis and function in TBH‐induced C2C12 myotubes..

### 2.4 PRDX5 was identified as target of MY mediating the mitochondria protective effect

**FIGURE 4:**
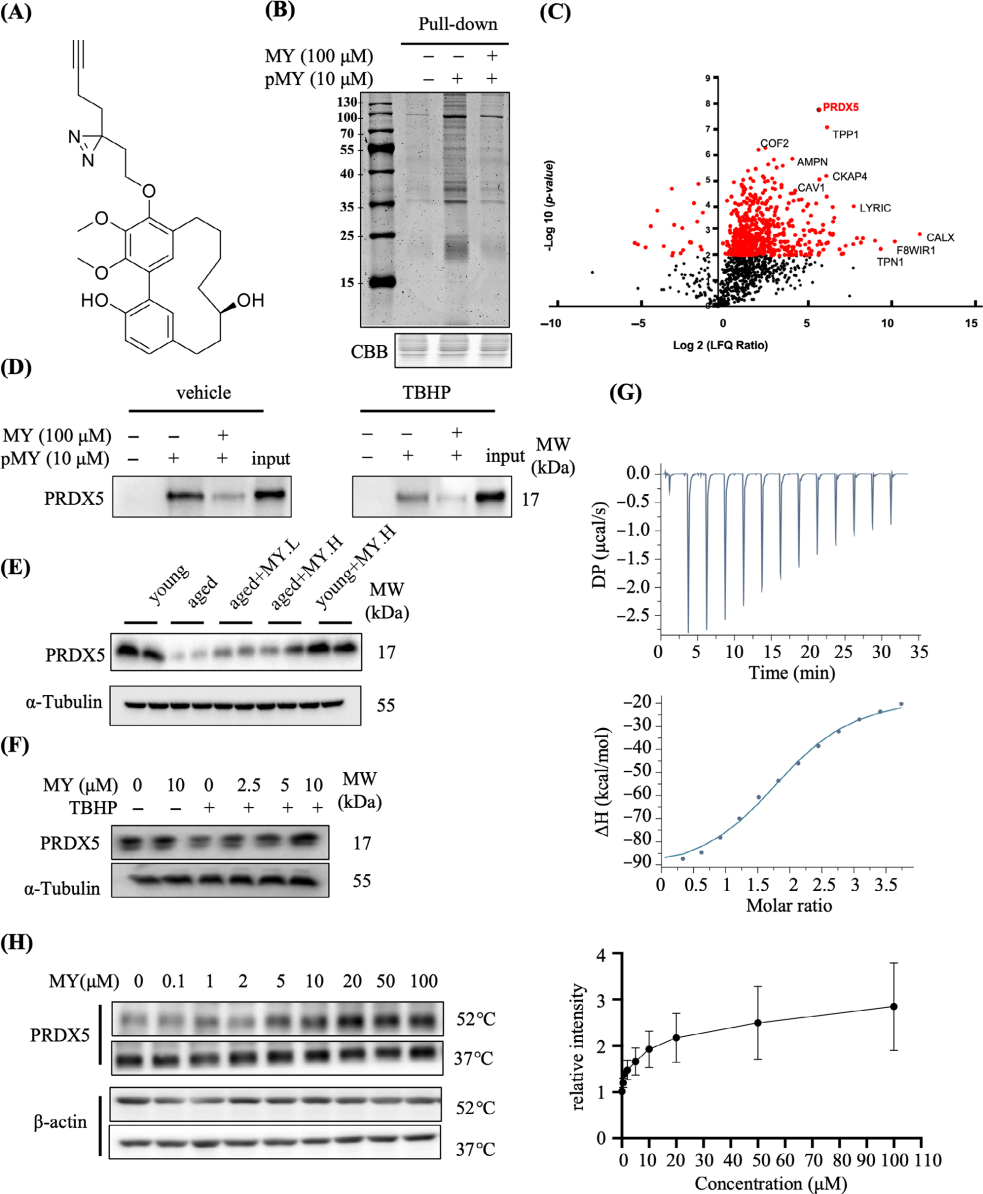
Target identification of MY in protecting oxidative damage in TBHP‐treated C2C12 myotubes. (A) The structure of the probe MY‐P. (B) Pull‐down labeling with MY‐P in C2C12 myotubes. CBB: Coomassie Brilliant Blue. (C) Volcano plot of MY‐P binding proteins compared with control group (10 µM MY‐P). (D) Pull‐down/Western blotting identified the target protein PRDX5. (E) The PRDX5 expression in Gast muscle from young or aged mice. ##*p *< 0.01, young versus aged. **p < 0.01, aged versus aged+MY.L. &&*p* < 0.01, aged versus aged+MY.H. (F) The PRDX5 expression in TBHP‐treated C2C12 myotubes. ##*p* < 0.01, vehicle versus TBHP, *p < 0.05, **p < 0.01, MY versus TBHP. (G) ITC titration of MY (100 µM) into recombinant PRDX5 protein (5 µM). (H) Western blotting‐based CETSA validation of thermal stabilization of PRDX5 in response MY treatment at the concentration from 0 to 100 µM. β‐Actin was used as a control. Data are displayed as mean ± SD, *n* = 6.

To identify the protein targets of MY mediating the protective effect against oxidative damage in C2C12 myotubes, we synthesized a probe of MY containing a linker with a diazirine moiety and a terminal alkyne (MY‐P; Figure 4A). We first evaluated whether MY‐P retains the bioactivities of MY. As expected, MY‐P showed the similar effects in improving cell viability, reducing ROS production, and enhancing ATP content in TBHP‐treated C2C12 myotubes, as those of MY (Figure S4). Next, affinity‐based protein profiling and bioimaging approach was recruited to identify the targets of MY. C2C12 myotubes were incubated with 10 µM MY‐P, 10 times excessive the parental competitor, MY, or DMSO to distinguish drug targets from nonspecific binding. As presented in Figure 4B, MY‐P displayed potent labeling efficacy and produced obviously visible bands at 10 µM. The labeling profiles of 10 µM MY‐P were much weaker with the MY at 100 µM. The result indicated that MY‐P bind similar targets to MY (Figure 4B). The click reactions combined with cellular imaging were carried out to investigate the distribution of MY‐P protein targets within C2C12 cells. The protein targets of MY‐P were mainly localized in cytoplasm in C2C12 myotubes (Figure S5). These data suggested that MY‐P had a high bioconjugative potency under in situ conditions. The MY‐P could be confirmed as a proper probe for chemical proteomics assays.

Subsequently, large‐scale pull‐down/LC–MS experiments resulted in the identification of a series of potential protein targets. The top 10 highest LFQ ratio hits with (log2 LFQ_Ratio) > 5 were labeled in the plot. Among the complete list of the identified proteins, PRDX5 showed the most significant potential, which was identified as one of the direct protein targets of MY. Moreover, according to the KEGG annotations, pathways including peroxisome (K11187), protein processing in endoplasmic reticulum (K13999), and endocytosis (K06278) participate in the regulation. Among these genes, peroxisome is closely related to the redox homeostasis. PRDX5 is important in the antioxidant system involved in hydrogen peroxide metabolism. Combined with the *p* value and the logFC values, PRDX5 is considered as the key factor mediating MY's effect in alleviating ROS accumulation. As shown in Figure 4D, MY‐P could pull down PRDX5, which was competed away by a high dose of MY. Interestingly, the protein level of PRDX5 was decreased in muscle from aged mice compared with young mice and in TBHP‐treated C2C12 myotubes, and MY treatment restored PRDX5 expression level (Figures 4E and F). To confirm the direct interaction between MY and PRDX5, the isothermal titration calorimetry (ITC) assay was carried out to detect the dynamic characteristics. The results showed that MY binds to PRDX5 complex with a 1:1 stoichiometry, with a *K*_d_ value of 1.08 ± 0.30 µM, and that the binding between MY and PRDX5 is strongly enthalpy‐driven (Δ*G* = −8.51 kcal/mol, −*T*Δ*S* = −15.4 kcal/mol, Δ*H* = −80 kcal/mol), suggesting that hydrogen bonds and electrostatic interactions might play key roles in the binding of MY to PRDX5 (Figure 4G). Furthermore, cellular thermal shift assay (CETSA)‐Western blotting assay was performed to evaluate the thermostable property of the MY–PRDX5 complex. As shown in Figure 4H, MY treatment increased the thermostability of PRDX5 in a dose‐dependent manner at the concentration from 0 to 100 µM compared with the control group. Thus, PRDX5 might be a direct target of MY in myotubes.

### 2.5 MY protects C2C12 myotubes against oxidative damage through targeting PRDX5

**FIGURE 5:**
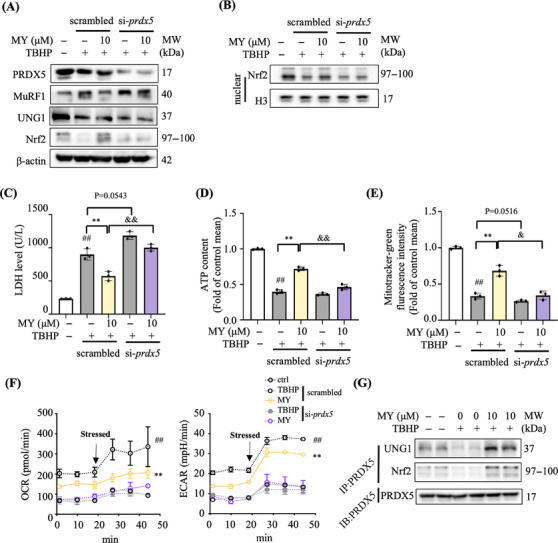
MY protects C2C12 myotubes against oxidative damage through targeting PRDX5. (A) Protein expression levels of PRDX5, MuRF1, UNG1, and Nrf2 in si‐*Prdx5* and scrambled cells. β‐Actin was used as a loading control. (B) Protein expression level of nuclear Nrf‐2 in si‐*Prdx5* and scrambled cells. Histone H3 was used as the loading control. LDH level (C) and ATP concentration (D) in TBHP‐treated scrambled and si‐*Prdx5* cells. (E) Mitochondrial content in TBHP‐treated scrambled and si‐*Prdx5* cells, assessed by MitoTracker Green staining. (F) Oxygen consumption rate (OCR) and extracellular acidification rate (ECAR) in TBHP‐treated scrambled and si‐*Prdx5* cells, assessed by Seahorse assay. (G) The levels of coprecipitated Nrf2 and UNG1 with PRDX5 in C2C12 myotubes. Data are displayed as mean ± SD, *n* = 6. #*p* < 0.05, ##*p* < 0.01, vehicle versus TBHP. *p < 0.05, **p < 0.01, TBHP+MY versus TBHP. &*p* < 0.05, &&*p* < 0.01, si‐*Prdx5*+MY versus scrambled+MY.

The expression level of PRDX5 protein was around 60% less in si‐*Prdx5* cells than that in the scrambled siRNA transfected cells (scrambled) (Figures 5A and S6). Strikingly, MY treatment induced decrease of MuRF1 expression and increase of UNG1 and Nrf2 expression, as well as promoted nuclear accumulation of Nrf2 protein, the above effects were totally abolished in si‐*Prdx5* cells (Figures 5A and B). In addition, MY failed to reverse TBH‐induced increase of LDH level and reduction of mitochondrial content and ATP production in si‐*Prdx5* cells (Figures 5C‒E). Under basal status and carbonyl cyanide‐p trifluoromethoxyphenylhydrazone (FCCP) stimulated condition, the curves underlied that TBHP‐treated myotubes showed weaken mitochondrial respiration, while MY treatment rescued oxygen consumption rate (OCR) suppression induced by TBHP (Figure 5F). The glycolysis was enhanced by MY administration in TBHP‐exposed myotubes as revealed by higher level of extracellular acidification rate (ECAR) (Figure 5F). However, the MY protective effects on ECAR and OCR were diminished in si*Prdx5* cells as shown in Figure 5F. The coimmunoprecipitation data showed that fewer levels of UNG1 and Nrf2 were pulled‐down by PRDX5 after TBHP treatment, which was reversed by MY treatment (Figure 5G) compared with the control cells. Thus, MY protects oxidative damage‐induced mitochondrial dysfunction in myotubes mainly through targeting PRDX5.

### 2.6 Cys100 of PRDX5 is critical for binding to MY

**FIGURE 6:**
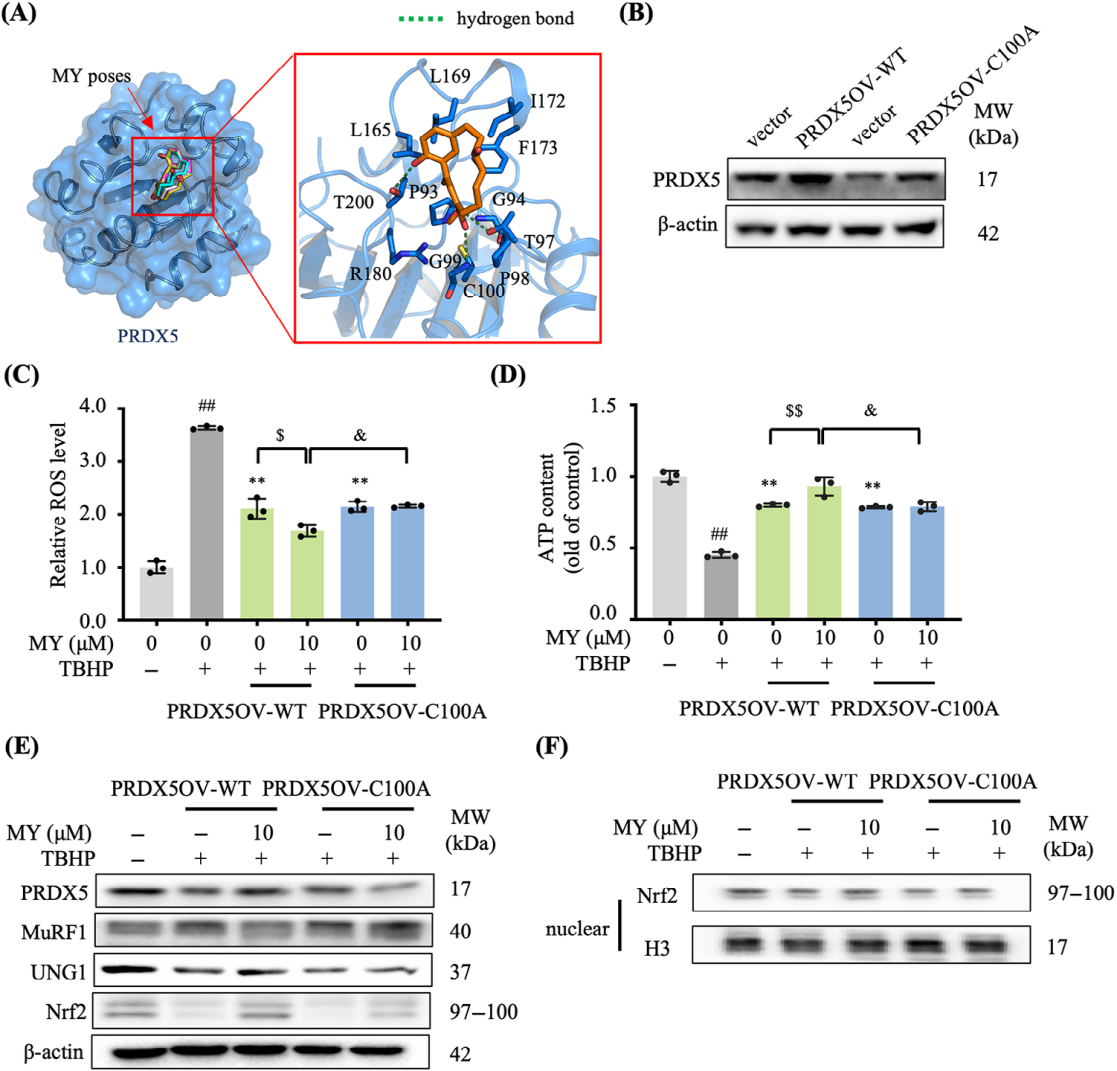
Cys100 of PRDX5 is critical for binding to MY. (A) Binding sites of MY–PRDX5 by virtual docking analysis. (B) The PRDX5 expression level in PRDX5‐WT and PRDX5‐C100A overexpression C2C12 cell lines, respectively. (C) ROS production. (D) ATP concentration. (E) Protein expression levels of MuRF1, UNG1 and Nrf2 in TBHP‐treated PRDX5‐WT and PRDX5‐C100A overexpression cells. β‐Actin was used as a loading control. (F) Protein expression level of nuclear Nrf‐2 in TBHP‐treated PRDX5‐WT and PRDX5‐C100A overexpression cells. Histone H3 was used as the loading control. Data are shown as mean ± SD, *n* = 6. #*p* < 0.05, ##*p* < 0.01, vehicle versus TBHP. *p < 0.05, **p < 0.01, TBHP+MY versus TBHP. &*p* < 0.05, &&*p* < 0.01, WT+MY versus C100+MY. $*p* < 0.05, $$*p* < 0.01, TBHP versus MY+TBHP.

Interestingly, molecular docking analysis showed that MY fitted well in the binding pocket of PRDX5 (Figure 6A). Superimposed of the top‐5 high score conformations of MY binding to PRDX5 indicated the poses of MY in PRDX5 is stable. The interface of MY and PRDX5 involved hydrogen bonding with T97, G99, T200, and hydrophobic interactions of C100, L165, L169, I172, and F173 (Figure 6A).

The site‐directed mutagenesis was employed to confirm if the Cys100 of PRDX5 is involved in the interaction between MY and PRDX5. The wild‐type PRDX5 (PRDX5‐WT) and site‐directed mutagenesis of the Cys100 residue of PRDX5 to an alanine residue (PRDX5‐C100A) overexpressed C2C12 cell lines were generated, respectively (Figure 6B). As shown in Figures 6C and D, mutation of Cys100 led to a failure of MY to enhance the cells’ resistance to oxidative stress by promoting the ROS scavenging and ATP production. Consistently, the Western blotting data indicated that MY treatment induced increases of UNG1 and Nrf2 and nuclear accumulation of Nrf2 protein, and decrease of MuRF1, which were almost abolished in TBHP‐treated PRDX5‐C100A overexpression cells (Figures 6E and F). The protective effects of MY were only present in the TBHP‐treated PRDX5‐WT overexpression cells, but not in the TBHP‐treated PRDX5‐C100A overexpression cells, demonstrating the Cys100 residue is crucial for the MY–PRDX5 interaction.

## 3 DISCUSSION

During aging, excessive ROS production in skeletal muscle results in redox imbalance with dysfunction of mitochondria and oxidative damage, leading to muscle mass loss and dysfunction.^22^ It is suggested that interventions that reversing oxidative stress‐induced mitochondrial dysfunction could protect against sarcopenia.^23^ However, clinically effective therapies against sarcopenia are not available except for protein supplements and exercise, which are difficult to maintain in the elderly.^24^ In the current study, a naturally occurring small molecule, MY, was found to mitigate aging‐associated loss of muscle mass and strength, through protecting mitochondria against oxidative damage, which might be further developed as a candidate to treat sarcopenia.

TBHP exposure has been shown to result in oxidative stress via degradation of L‐Opa1 and increase in Mfn1, leading to muscle damage.^25^ The cell viability as well as ATP production of C2C12 myotubes administrated with indicated concentrations of TBHP were evaluated. High concentration of TBHP (400 µM or higher) caused severe cell death, in turn decreasing ROS production (Figure S3). At a concentration of 200 µM, TBHP only caused slightly cell death and induced ROS production maximally; thus, this concentration of TBHP was chosen in the in vitro studies (Figure S3).

To remove and consequently repair delaminated, oxidized, and alkylated DNA bases, the base excision repair pathway is employed.^26^ The DNA glycosylases cut the damaged nucleotide base. Then, this special enzyme catalyzed and initiated this highly coordinated repair pathway. Among them, UNG1 is normally excises uracil from DNA. Current study found that MY decreases 8‐OH‐dG level and increases the expression of UNG1 in skeletal muscle from aged mice and TBHP‐treated C2C12 myotubes, indicating reduced mtDNA injury, which in turn prevent against mitochondrial dysfunction.

PRDX5 is one of the critical regulators of mitochondrial hydrogen peroxide. Our study indicated MY directly binds to PRDX5 to protect myotubes against oxidative damage and mitochondrial dysfunction. These evidence suggested that PRDXs could be potential therapeutic targets to treat and/or prevent sarcopenia. A PRDX5 knockout or overexpression mice model should be set up to elucidate the role of PRDX5 in aging associated muscle wasting.

Studies have focused on this conserved motif and an arginine distant from PRDXs sequences complexed with H_2_O_2_, the arginine residue is pivotal in delivering the oxygen of the peroxide, disrupting the O─O bond and stabilizing transition between the motif and the proximal O.^15^ Thus, understanding of the activation mechanism of perodiredoxins is potential in novel drug design. In PRDX5, the motif sequence is P_93_GAFTPGC_100_, and our data indicated that Cys100 residue of PRDX5 is critical for the interaction between MY and PRDX5. It is known that formation of an intramolecular disulfide bond between the sulfhydryl groups of Cys100 and Cys125 in oxidative environment is necessary for PRDX5 activation. Due to the hydrophobic prosperity, MY possesses higher affinity and is prior to interact with the core region of PRDX5 around the active stie Cys100. From the docking results, MY forms hydrophobic interactions with Cys100, which possibly displays an allosteric modulation of PRDX5 by alter the orientation of the sulfhydryl group of Cys100, making the distance with sulfhydryl group of Cys125 being closer, as well as their dihedral angle approximately 180°. Thus, with the presence of MY, PRDX5 is prone to form an intramolecular disulfide bond, leading to activation of PRDX5. Based on the structure of PRDX5 protein, structure‐guided chemical modification of MY may suggest more potent candidate molecules with higher bioactivity and less adverse effects for clinical usage.

It is yet to report the interaction between UNG1 and PRDX5. We proposed that the interaction might stabilize the conformation of UNG1 and enhance its DNA repair enzymatic activity. UNG is important in DNA repair in both nucleus and mitochondria. UNG1 overexpression was shown to strengthen cell resistance against oxidative stress without alterations in cell morphology.^27^ It was reported that UNG1 binds to PRDX3 via a disulfide linkage to protect UNG1 from ROS‐mediated degradation and prevent mtDNA against oxidative stress.^27^ The coimmunoprecipitation study indicated that PRDX5 recruits UNG1 and Nrf2 under oxidative stress, alleviating the local concentration of antioxidant enzymes, leading to scavenging ROS and protection of mtDNA against oxidative damage and enhance the mitochondrial function and biogenesis.

Pull‐down assay coupled with labeling/Western blotting/LC–MS/MS were performed in the current study to discover PRDX5 as a direct target of MY in myotubes. Herein, CETSA assay and ITC assay were used to verify the interaction between MY and PRDX5. Further studies, such like cocrystal structure of PRDX5 with MY, are needed to fully elucidate the mode of action of MY on PRDX5.

PRDXs are much more abundant than GPxs by constituting around 0.1–0.8% of total soluble protein in the cells.^28^ Thus, PRDXs are prime candidates for mediating hydrogen peroxide signaling. Notably, MY is shown to increase the expression of PRDX5 in TBHP exposed myotubes and skeletal muscle from aged mice. Nrf2 is required for the maintenance of high PRDX5 gene expression under oxidative stress.^29^ PRDX5 interacts with Nrf2 to participate in its nuclear translocation and enhance its antioxidative capacity.^30^ Previously, quercetin has been shown to induce Nrf2 expression, transactivating the promoter activity of both PRDX3 and PRDX5, but the activation was completely abolished by knockdown of Nrf2.^31^ The cellular redox activity responded to excessive ROS is found to mediate by eliciting the adaptive transcriptional program controlled by Nrf2, thus enhanced the antioxidant genes expression like PRDX5.^32^ PRDX5 and Nrf2 might form a loop to interact with each other to enhance antioxidative capacity under oxidative stress conditions.

## 4 CONCLUSIONS

**FIGURE 7:**
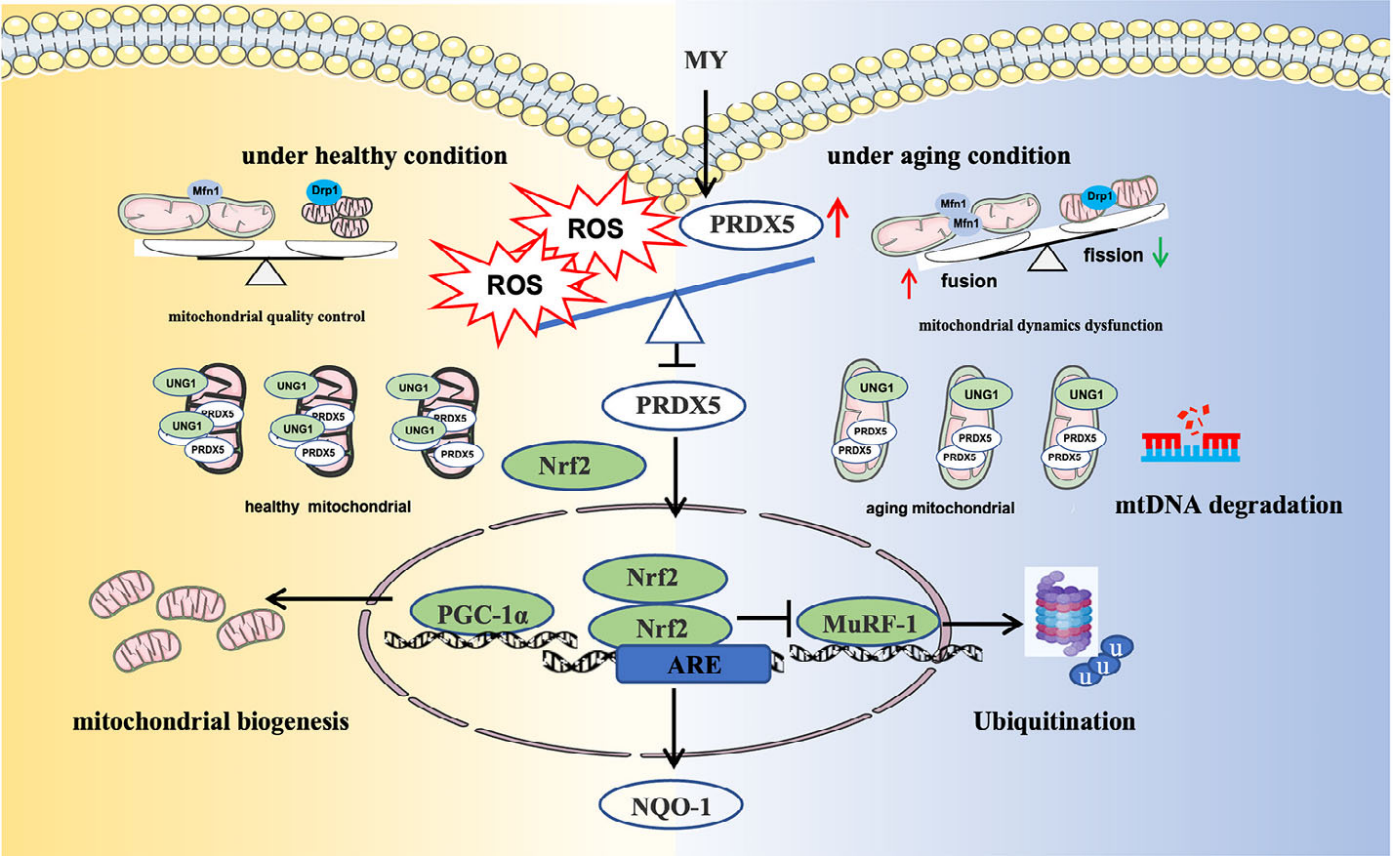
Schematic diagram of molecular mechanism of MY in mitigating sarcopenia.

MY possesses protective effect against aging‐associated skeletal muscle wasting through mitigating oxidative stress‐induced mitochondrial dysfunction (Figure 7). Additionally, MY directly binds to Cys100 of PRDX5. Our study provides a drug candidate for treating sarcopenia and supplies a target for screening small molecules against sarcopenia.

## 5 MATERIALS AND METHODS

### 5.1 Animals

The experimental procedures of animal studies were approved by the China Animal Care and Use Committee and the Care and Use of Laboratory Animals of China Academy of Chinese Medical Sciences (No. 2021B025; Beijing, China).

### 5.2 Animal experimental procedure

Three‐ or eighteen‐month‐old male C57BL/6J mice were purchased from Vital River Laboratory Animal Technology (Beijing, China). The mice were maintained with free access to water and food with the temperature around 23 ± 1°C and 12 h light–dark cycle.

Twelve 3‐month‐old mice were distributed randomly into two groups (six mice per group). The mice of young group were injected with PEG 400 (30% in 0.9% saline, 10 mL/kg body weight). Mice in the other group were treated with equal volume of PEG 400 buffer containing high dose of MY (young+MY.H, 50 mg/kg MY). The life course approach to sarcopenia prevention is important, and intervention at middle‐age and earlier provides good opportunity.^33^ Some studies have proved the occurrence of sarcopenia phenotypes in mice at 18‐month‐old, and 18‐month‐old mice as aged model compared with that of the young mice at 2.5‐month‐old showed approximately 30% weight loss of Gast muscle.^34^ To express accurately, 18‐month‐old mice were considered as aged mice in the current study. Eighteen 18‐month‐old mice were divided into three groups (*n* = 6) randomly. In aged group, the mice were treated with PEG 400 solution as described in the young group. Mice of another two groups were injected by the equal volume of PEG 400 solution with low dose of MY (aged+MY.L, 10 mg/kg MY) and high dose of MY (aged+MY.H, 50 mg/kg MY), respectively. The mice were injected once a day intraperitoneally for 20 days. The grip strength and body weight were recorded every other day. Twenty‐four hours after the last injection, blood samples were collected from mice under anesthesia (inhalation 3% isoflurane gas at 0.5 L/min). The collected sera were stored at −80°C fridge. The Quad, TA, soleus, Gast, extensor digitorum longus, and the liver were dissected. The collected muscles were weighed and frozen in liquid nitrogen immediately. The rate reflected atrophy was determined by the ratio of muscle weight divided by body weight.

### 5.3 Histological analysis of skeletal muscle

A middle part of Gast was collected and fixed in 4% paraformaldehyde and embedded into paraffin; sections were cut into 4 µm diameter and used for H&E staining. A total of 20 views were picked randomly from each slide with muscle cut in the cross section under the microscope (Olympus, Tokyo, Japan). The diameters of all the muscle fiber were measured by the CellSens image analysis system (Olympus).

### 5.4 Grip‐strength test

The value of muscle strength was measured by an SA417 grip‐test meter system (SANS, Nanjing, China). Mice were controlled to hold on a metal grid with their paws. Then, the mice were gently pulled backward by holding their tail until they could not hold the grid. The peak pull force was obtained on a digital force transducer. The average value of five replicates of measurements was calculated and defined as the grip strength of the mouse.

### 5.5 Forced swimming test

The forced swimming test is one of the most commonly used models for evaluating the muscle damage properties in pharmacological studies.^35^, ^36^ In addition, the forced swimming test has been used to measure locomotor activity. The result is one of the indexes evaluation of muscle protective properties of the compounds in our previously reported studies.^18^, ^37^ Combining with the grip strength measurement, the swimming time is positive correlatedly to the muscle strength. The mice were loaded in the acrylic plastic pool containing fresh water. The water depth was approximately 30 cm. The swimming time of each mouse was recorded, exhaustion was determined as failure to rise its head to the water surface in 7 s and loss of coordinated movements.

### 5.6 Transmission electron microscopy analysis

The 1 mm^3^ of Gast muscle of each sample was collected and fixed in 2.5% glutaraldehyde. The samples were rinsed with 1 mM phosphoric acid solution. Next, 1% osmium tetroxide containing 50 mM potassium ferricyanide was used to fix, then embedded in epoxy resin. The blocks were cut carefully into ultrathin (50 nm) sections, and analyzed with a JEM 1200EX transmission EM (JEOL, Tokyo, Japan) at Zhongkebaice (Beijing, China).

### 5.7 Western blotting analysis

The cytoplasmic and nuclear proteins were isolated by a cytoplasmic and nuclear protein extraction kit (Beyotime, China), following the manufacturer's illustration, respectively. Protein concentration of each sample was determined using the BCA protein assay kit (Thermo‐Fisher, Grand Island, NY). Equal amount of proteins (20‒30 µg) were loaded on SDS‐PAGE gel, and then transferred to polyvinylidene fluoride membranes (Bio‐Rad, Hercules, CA). The membrane was blocked with 5% nonfat milk in TBST buffer for 1 h at room temperature. Specific primary antibody (Table S1) was blotted to the membrane overnight at 4°C. A horseradish peroxidase‐conjugated secondary antibody was used for blotting for 2 h at room temperature. A SuperSignal West Femto Maximum Sensitivity Substrate kit (Thermo, Rockford, IL, USA) was used to detect the immune‐blotting signal. The ChemiDoc MP Imaging System was employed to visualize the specific protein bands. The bands were quantitated with Image Lab 5.1 (Bio‐Rad).

### 5.8 Analysis of oxidative damage of mtDNA

To analyze the oxidative products of mtDNA, the levels of 8‐OH‐dG were measured using an ELISA kit (CSB‐E10527m; Cusabio, China). The mitochondria from Quad muscle were isolated using a Tissue Mitochondrial Isolation Kit (Beyotime, Shanghai, China). The mitochondria from C2C12 myotubes were isolated using a Cell Mitochondria Isolation Kit (Beyotime). All the procedures were conducted following the manufacturer's protocol. SpectraMax M5 microplate reader (Molecular Devices, CA) was utilized to record the absorbance at 450 nm.

### 5.9 Cell culture

The C2C12 myoblasts were acquired from ATCC. The cells were cultured in DMEM contained 10% fetal bovine serum and 1% Pen/Strep (P/S) in the incubator with 5% CO_2_ at 37°C. The cells were grown to 70−80% confluence and incubated with DMEM containing 2% heat inactivated HS and 1% P/S for 4 days in order to initiate differentiation. TBHP (200 µM) was used to treat the fully differentiated myotubes, with or without the existence of MY as indicated for 24 h. DMSO was treated as a vehicle control.

### 5.10 Cell viability and intracellular ROS production

Cell viability was determined by using Enhanced CCK‐8 kit according to the manufacturer's instruction. C2C12 cells were seeded at a density of 5 × 104 cells per well in 96‐well plates. The myotubes were treated with different concentrations of MY for 24 h with or without 200 µM TBHP after fully differentiated. Next, the cells were incubated for another 30 min after added 10 µL enhanced CCK‐8 solution to each well. SpectraMax M5 microplate reader (Molecular Devices) was utilized to record the absorbance at 450 nm. Cytotoxicity was further determined by the level of LDH in the cell culture medium using the LDH Release Assay Kit (Beyotime) according to the manufacturer's guideline.

The 2,7‐dichlorodihydrofluorescein diacetate (Beyotime) was used to measure the intracellular ROS levels as previously described.^38^ In brief, C2C12 myoblasts were seeded into each well of a black‐wall 96‐well plate with a transparent bottom. Fluorescence intensity was recorded by the flow cytometry (FACS Calibur; BD, Lake Franklin, NJ, USA).

### 5.11 Determination of H_2_O_2_ level

The cellular H_2_O_2_ level was investigated using a hydrogen peroxide assay kit following the manufacturer's manual (Beyotime). In brief, the harvested cells were lysed and centrifuged at 12,000×*g* for 5 min. Next, the cell lysate and test solution were incubated for 30 min at room temperature. The absorbance at 560 nm was recorded by a SpectraMax M5 microplate reader (Molecular Devices).

### 5.12 MitoROS determination

MitoSOX Red (Yeasen, Shanghai, China) was used to measure the superoxide levels of the TBHP‐treated C2C12 myotubes. Briefly, the cells were incubated with or without MY for 24 h then exposed with MitoSOX Red for 30 min. The cellular fluorescence intensity was measured by flow cytometry at excitation and emission wavelength at 510 and 580 nm, respectively (BD).

### 5.13 Immunofluorescence staining

4% (v/v) formaldehyde was employed to fix the fully differentiated C2C12 myotubes at room temperature for 20 min. 0.1% Triton X‐100 in PBS at room temperature for 1 h was used to permeabilize and block the fixed cells, after being washed with PBS twice. The cells were then incubated with MyHC antibody at 4°C overnight. The goat anti‐mouse secondary antibody tagged with the Alexa Fluor was used to block the cells for 2 h at room temperature. 4.5‐Diamidino‐2‐phenylindole (1 µg/mL) was employed for staining for 20 min. After extensive washing, the cells were mounted with vectashield and observed with a Nikon fluorescence microscope (Olympus IX71 Motorized Inverted Microscope, DP controller, Soft Imaging System; Olympus).

### 5.14 Determination of ATP content

An ATP content kit was utilized to measure the cellular ATP content through the manufacturer's protocol (Beyotime). The luminescence intensity was recorded with a luminometer (SpectraMax M5). Protein content was set as reference to normalize the ATP levels. The data were expressed as relative ATP levels compared with the control.

### 5.15 Seahorse analysis

Seahorse XF Cell Energy Phenotype Test Kit (103325‐100; Agilent Technologies, Santa Clara, CA) was used to measure the OCR and ECAR on a Seahorse Bioscience XF24‐3 Extracellular Flux Analyzer (Agilent Technologies). C2C12 cells were seeded and after fully differentiated, the cells were incubated in XF assay medium and treated with or without MY as indicated. Next, the cells were maintained in the absence of CO_2_ for 1 h. To measure the OCR and ECAR under stressed condition, 1 µM oligomycin and 1 µM FCCP were introduced in real time, after measuring basal OCR and ECAR condition. The data were normalized by cell number. Cell number was determined by using Enhanced Cell Counting Kit‐8 (CCK‐8; Beyotime).

### 5.16 Protein target identification and validation

To identify the protein targets of MY, pull‐down experiment was carried out, followed by labeling, Western blotting, and LC‐MS/MS, where applicable. The MY probe (MY‐P) was synthesized in our previous study.^39^ Fully differentiated C2C12 myotubes were washed twice with PBS and treated with probe‐containing medium (the final concentration of MY‐P was 10 µM) with the absence or presence of 10× MY for 2 h. The cells were exposed to 365 nm UV light for 20 min before lysed with RIPA lysis buffer on ice. The harvested cells were briefly sonicated before centrifugation. The reactions were quenched by adding 8 volumes of ice‐cold methanol and placed at −20°C for 30 min to precipitate proteins. The protein pellet was air‐dried and then resuspended in PBS with 1% SDS by sonication. The peptides eluted from the MonoSpin C18 were dried down by SpeedVac. The obtained samples were ready for LC–MS/MS analysis.

The peptides were detected using an Orbitrap Fusion Lumos mass spectrometer (Thermo Fisher) as previously described.^39^ The 20 most intense ions above a 1000 counts threshold were picked for fragmentation by HCD with a maximum ion accumulation time of 50 ms. Isolation width of 1.6 *m/z* units was used for MS2. Single and unassigned charged ions were excluded from MS/MS. For HCD, normalized collision energy was set to 28%.

The raw data were searched against MaxQuant 2.1.0.0 with MS tolerance of 4.5 ppm, and MS/MS tolerance of 20 ppm. The hits were blast searched against the UniProt mouse protein database (release 2022_08, 86,436 sequences). Label‐free quantification was employed to quantify the protein abundances.

### 5.17 Molecular docking

The structure of PRDX5 (PDB ID: 3MNG) was prepared for docking analysis, using the Autodock tool.^40^, ^41^ Hydrogen atoms were added before docking analysis in the Python Molecular Viewer (v 1.5.7). After ligands preparation, dockings of MY to PRDX5 was performed with the AutoDock tool with minor change of the parameters.^19^ The default settings of docking parameters were used. The Lamarckian genetic algorithm was used for docking process. The binding conformations selected from the optimal poses was presented for the binding sites of MY and PRDX5.

### 5.18 RNA interference

The siRNA targeting *Prdx5* (#1080, sense: GCUACCCAGAUAACUUUCUTT; antisense: AGAAAGUUAUCUGGGUAGCTT) was purchased from Santa Cruz. Scrambled non‐targeting siRNA (sc‐37007; Santa Cruz) was used as a negative control. C2C12 cells were seeded into six‐well plates at a concentration of 5 × 105 cells per well. After differentiation, C2C12 myotubes were transfected with 80 nM si‐*Prdx5* or scrambled siRNA using Lipofectamine 3000 transfection reagent (Invitrogen) and incubated at 37°C for 6 h according to the manufacturer's recommendations. Subsequently, cells were switched to fresh medium and incubated for an additional 24 h.

### 5.19 Cellular thermal shift assay

Differentiated C2C12 cells were pretreated with MY (0‒100 µM) for 24 h. The cell lysates were collected for the CETSA measurements, 52°C was picked as the heating temperature, then 37°C as protein level control was obtained to control the applied compounds would not cause global distortion. The soluble proteins were analyzed by Western blotting.

### 5.20 Isothermal titration calorimetry

ITC experiments were carried out on a MicroCal PEAQ‐ITC (Malvern Panalytical). The protein sample, complexed by MY and recombinant PRDX5 protein (synthesized by ChinaPeptides, Shanghai, China), was dialyzed into the ITC buffer overnight. 100 µM MY was titrated against 5 µM PRDX5 protein. The titration was injected 13 times composed of 2 µL MY. The flow rate was set as 2 s/µL within 150 s. The test was performed at 25°C and stirred at 750 rpm. The collected data were processed to fit to the model of one single binding site using the Setup MicroCal PEAQ‐ITC Analysis Software.

### 5.21 Coimmunoprecipitation

Cell lysates were mixed with the indicated antibody and subsequently 20 µL protein A/G‐agarose beads (Santa Cruz Biotechnology) and incubated on a rotator for 4 h at 4°C. The beads were washed twice with PBS and then twice with lysis buffer supplemented with complete mini protease inhibitor cocktail. Bound proteins were boiled in sample preparation buffer for 5 min and then used for Western blotting analysis.

### 5.22 Generation of PRDX5 overexpression cell lines

The pcDNA3.1‐PRDX5 plasmid and pcDNA3.1‐PRDX5‐C100A plasmid were purchased from IGE Biotechnology (Guangzhou, China). The sequences of both plasmids were shown in Table S2. C2C12 cells (1 × 105 per well) were seeded in six‐well plate and cultured for 24 h. The cells were transfected with 1 µg plasmids (pcDNA3.1, pcDNA3.1‐PRDX5, or pcDNA3.1‐PRDX5‐C100A) using Lipofectamine 3000 reagent. 24 h after transfection, 800 µg/mL G418 was added to select positive cells for 2 weeks. All survival C2C12 cells were pooled together for further experiments.

### 5.23 Data and analysis

Group size is the number of independent values, and data were expressed as mean ± SEM based on at least three independent experiments and analyzed on GraphPad Prism 7 (GraphPad Software, San Diego, CA, USA). Statistical analysis was undertaken only for studies where each group size was at least *n* = 5. The significance of differences between groups was assessed by one‐way ANOVA followed by Tukey's post hoc test. *p* < 0.05 was considered statistically significant.

## AUTHOR CONTRIBUTIONS

Ligen Lin and Jigang Wang designed the study. Shengnan Shen and Qiwen Liao conducted the experiments and acquired data. Peng Lyu carried out LC–MS/MS experiments and conducted MS data analysis. Shengnan Shen and Qiwen Liao drafted the manuscript. Ligen Lin, Jigang Wang, Shengnan Shen, and Qiwen Liao revised the article. All authors read and approved the manuscript.

## CONFLICT OF INTEREST STATEMENT

The authors declare no conflict of interest.

## ETHICS STATEMENT

All animal experimental procedures were approved by the China Animal Care and Use Committee and the Care and Use of Laboratory Animals of China Academy of Chinese Medical Sciences (approval number 2021B025).

## Supporting information

Supporting Information

## Data Availability

The datasets used and/or analyzed during the present study are available from the corresponding author on reasonable request.
