# Supplementary material for: Myricanol prevents aging‐related sarcopenia by rescuing mitochondrial dysfunction via targeting peroxiredoxin 5
Source: MedComm (2020). 2024 Jun 12;5(6):e566. doi: 10.1002/mco2.566 (PMC11167181; doi:10.1002/mco2.566)
Supplement: Supplementary file 1 — Supporting Information [file MCO2-5-e566-s001.docx]

Supporting Information for

**Myricanol prevents ageing related sarcopenia by rescuing mitochondrial dysfunction via targeting peroxiredoxin 5**

Running title: Anti-Sarcopenia role of myricanol targeting peroxiredoxin-5

Shengnan Shen ^a,b,1^, Qiwen Liao ^b,1^, Peng Lyu ^b^, Jigang Wang ^a,d,e,*^, Ligen Lin ^b,c,*^

^a^ *State Key Laboratory for Quality Ensurance and Sustainable Use of Dao-di Herbs, Artemisinin Research Center, and Institute of Chinese Materia Medica, China Academy of Chinese Medical Sciences, Beijing, China*

^b^ *State Key Laboratory of Quality Research in Chinese Medicine, Institute of Chinese Medical Sciences, University of Macau, Macau*

^c^ *Department of Pharmaceutical Sciences and Technology, Faculty of Health Sciences, University of Macau, Macau*

*^d^ Shenzhen Institute of Respiratory Disease, Shenzhen People's Hospital (First Affiliated Hospital of South University of Science and Technology of China and Second Affiliated Hospital of Jinan University, China), Beijing, China*

^e^ *Department of Oncology, the Affiliated Hospital of Southwest Medical University, Luzhou, China*

^1^ These authors contribute equally to this work.

^*^Corresponding authors: Prof. Ligen Lin (ligenl@um.edu.mo); Prof. Jigang Wang (jgwang@icmm.ac.cn)

**
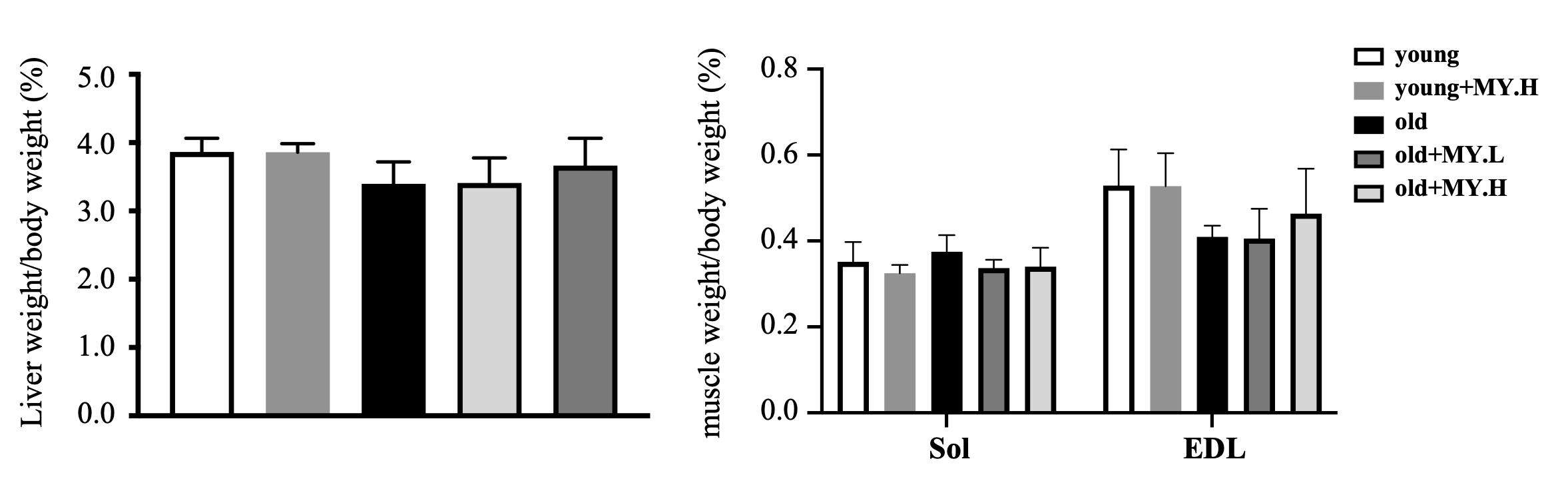
**

Figure S1 The ratios of liver, Sol and EDL to body weight. Data are shown as mean ± S.D., n = 6.

*
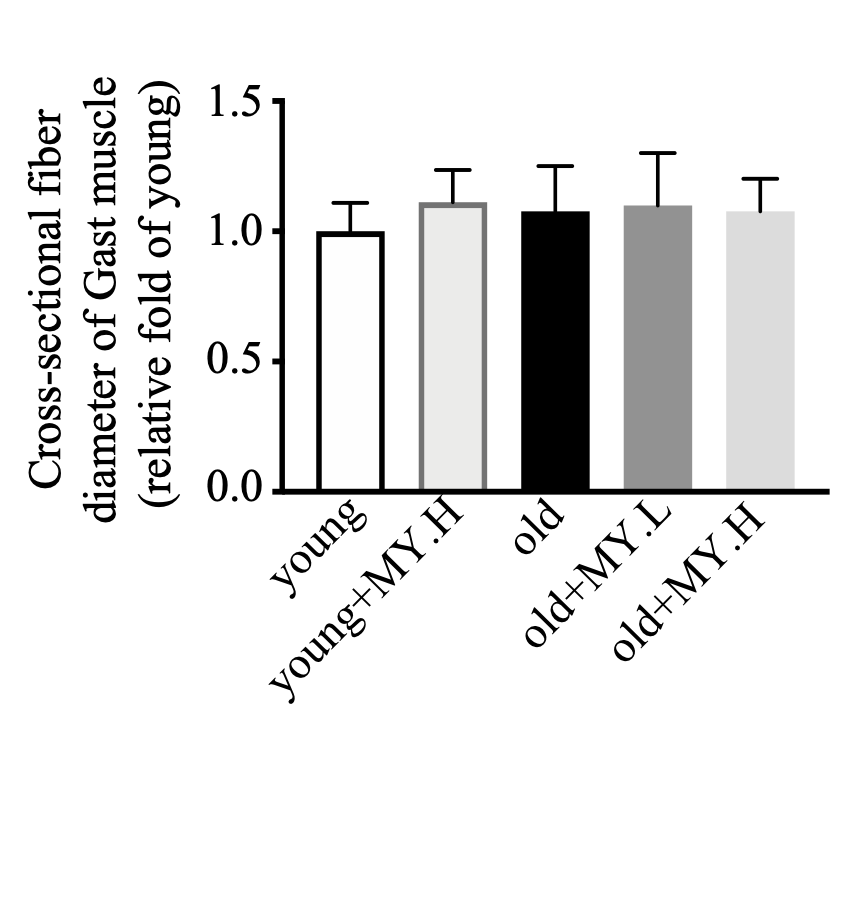
*

Figure S2 The diameter of muscle fiber in the cross-sectional and longitudinal-section, respectively. Data are shown as mean ± S.D., n = 6.


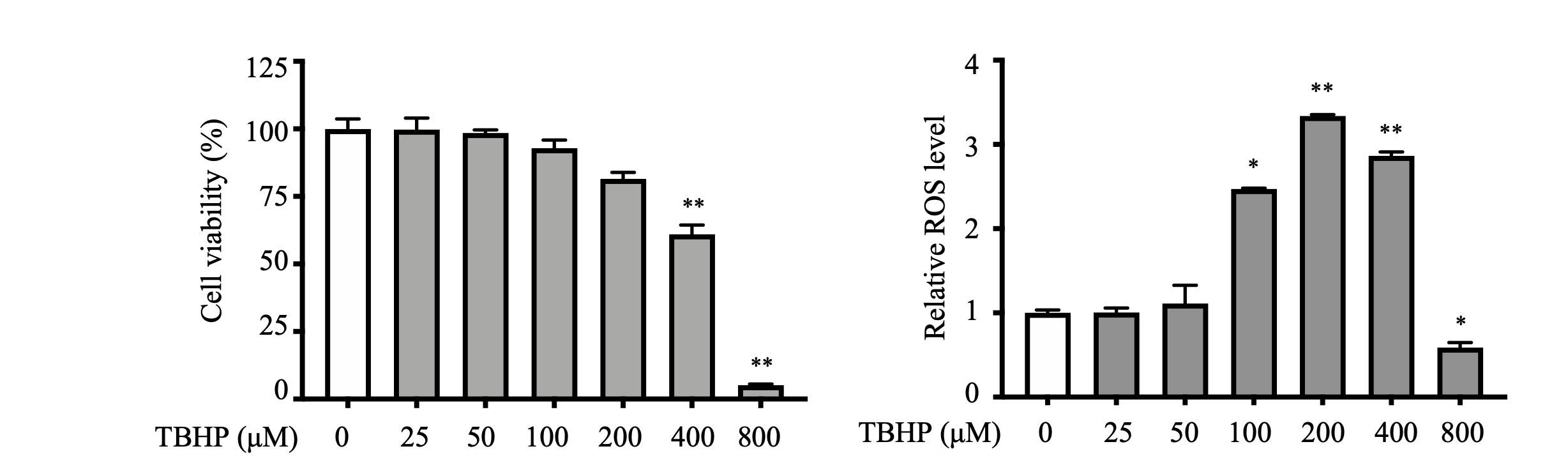


Figure S3 Cell viability and the ROS level in C2C12 myotubes treated with different concentrations of TBHP (25 to 800 μM). Data are shown as mean ± S.D., n = 6. **P* < 0.05, ***P* < 0.01, ctrl vs. TBHP.


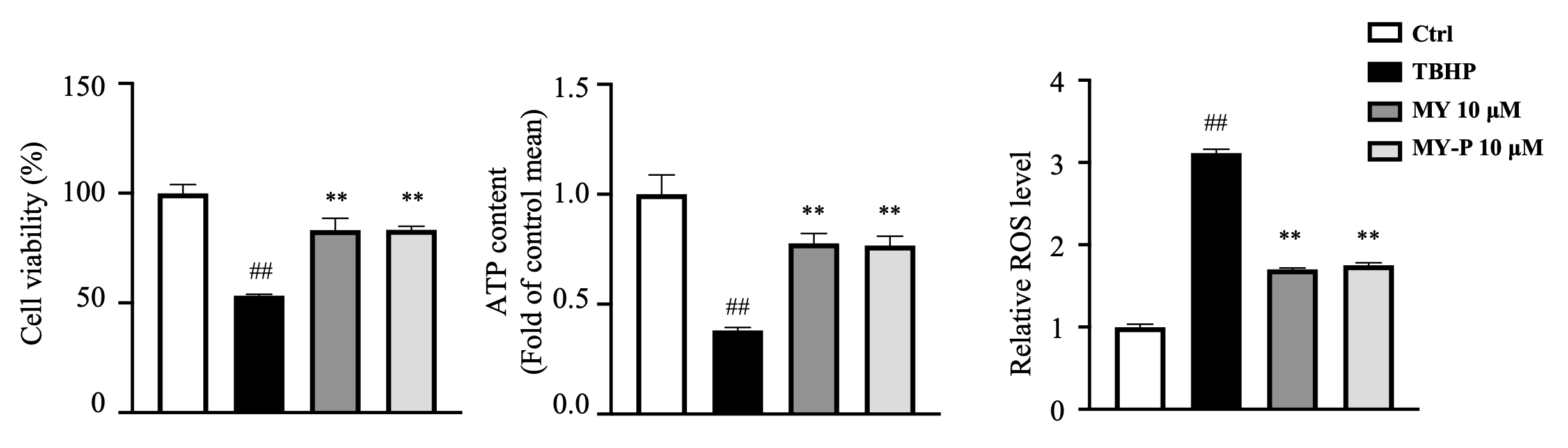


Figure S4 Cell viability, the ROS level and ATP concentration in TBHP-treated C2C12 myotubes treated with or without MY and MY-P. Data are shown as mean ± S.D., n = 6. ##*P* < 0.01, ##*P* < 0.01, ctrl vs. TBHP; ***P* < 0.01, MY or MY-P vs. TBHP.


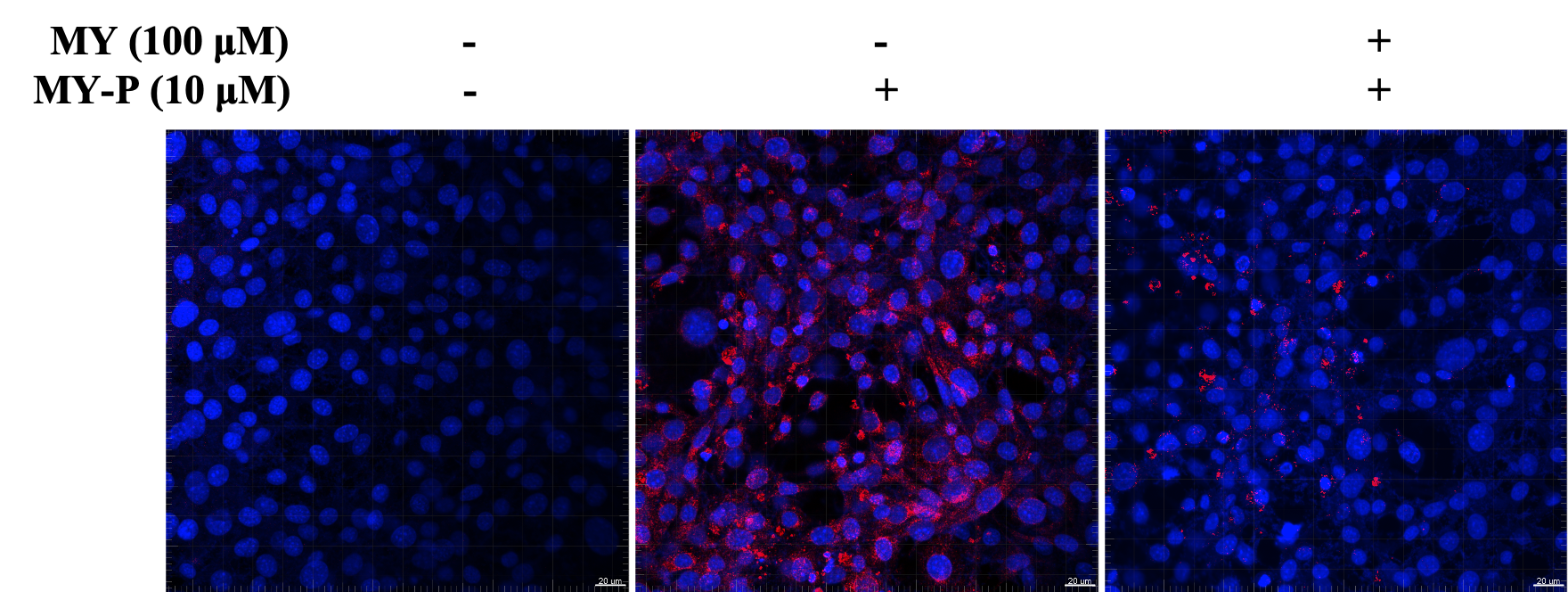


Figure S5 MY-P is mainly localized in cytoplasm in C2C12 myotubes. The labeling profiles of MY-P was almost abolished in the presence of MY at 100 μM.


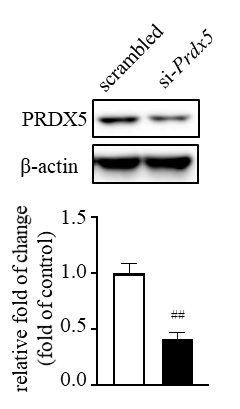


Figure S6 PRDX5 protein expression in scrambled and si-*Prdx5* C2C12 cells.

Table S1

Antibodies for immunoblotting.

| Antibody | Source | Vendor | Catalog No. |
| --- | --- | --- | --- |
| TRIM63 (MuRF1) | Rabbit | Proteintech | 55456-1-AP |
| MyOD | Mouse | Santa Cruz Biotechnology | sc-377460 |
| Myogenin | Mouse | Santa Cruz Biotechnology | sc-12732 |
| PGC-1α | Rabbit | Cell Signaling Technology | #2187 |
| β-actin | Rabbit | Santa Cruz Biotechnology | sc-1616 |
| Mfn1 | Rabbit | Santa Cruz Biotechnology | SC-50330 |
| Drp1 | Rabbit | Cell Signaling Technology | #8570 |
| p-Drp1(Ser637) | Rabbit | Cell Signaling Technology | #4867 |
| Nrf2 | Rabbit | Cell Signaling Technology | #12721 |
| Histone H3 | Rabbit | Cell Signaling Technology | #9715 |
| UNG1 | Rabbit | Abcam | ab47680 |
| NQO1 | Mouse | Proteintech | 67240-1-AP |
| PRDX5 (PRX5) | Rabbit | Proteintech | 17724-1-AP |
| PRDX5 (Peroxiredoxin5) | Rabbit | Abcam | ab180587 |
| MyHC | Mouse | R&D Systems | MAB4470 |

Table S2

Sequence of PRDX5 and its mutant

| Gene | Nucleotide sequence |
| --- | --- |
| PRDX5-WT | GGATCCCTTCCGCAGGGTGTCGCCGCTGTGCCGCTAGCGGTGCCCCGCCTGCTGCGGTGGCACCAGCCAGGAGGCGGAGTGGAAGTGGCCGTGGGGCGGGTATGGGACTAGCTGGCGTGTGCGCCCTGAGACGCTCAGCGGGCTATATACTCGTCGGTGGGGCCGGCGGTCAGTCTGCGGCAGCGGCAGCAAGACGGTACAGTGAAGGAGAGTGGGCGTCTGGCGGGGTCCGCAGTTTCAGCAGAGCCGCTGCAGCCATGGCCCCAATCAAGGTGGGAGATGCCATCCCAGCAGTGGAGGTGTTTGAAGGGGAGCCAGGGAACAAGGTGAACCTGGCAGAGCTGTTCAAGGGCAAGAAGGGTGTGCTGTTTGGAGTTCCTGGGGCCTTCACCCCTGGATGTTCCAAGACACACCTGCCAGGGTTTGTGGAGCAGGCTGAGGCTCTGAAGGCCAAGGGAGTCCAGGTGGTGGCCTGTCTGAGTGTTAATGATGCCTTTGTGACTGGCGAGTGGGGCCGAGCCCACAAGGCGGAAGGCAAGGTTCGGCTCCTGGCTGATCCCACTGGGGCCTTTGGGAAGGAGACAGACTTATTACTAGATGATTCGCTGGTGTCCATCTTTGGGAATCGACGTCTCAAGAGGTTCTCCATGGTGGTACAGGATGGCATAGTGAAGGCCCTGAATGTGGAACCAGATGGCACAGGCCTCACCTGCAGCCTGGCACCCAATATCATCTCACAGCTCTGAGGCCCTGGGCCAGATTACTTCCTCCACCCCTCCCTATCTCACCTGCCCAGCCCTGTGCTGGGGCCCTGCAATTGGAATGTTGGCCAGATTTCTGCAATAAACACTTGTGGTTTGCGGCCAGAATTC |
| PRDX5-C100A | GGATCCCTTCCGCAGGGTGTCGCCGCTGTGCCGCTAGCGGTGCCCCGCCTGCTGCGGTGGCACCAGCCAGGAGGCGGAGTGGAAGTGGCCGTGGGGCGGGTATGGGACTAGCTGGCGTGTGCGCCCTGAGACGCTCAGCGGGCTATATACTCGTCGGTGGGGCCGGCGGTCAGTCTGCGGCAGCGGCAGCAAGACGGTACAGTGAAGGAGAGTGGGCGTCTGGCGGGGTCCGCAGTTTCAGCAGAGCCGCTGCAGCCATGGCCCCAATCAAGGTGGGAGATGCCATCCCAGCAGTGGAGGTGTTTGAAGGGGAGCCAGGGAACAAGGTGAACCTGGCAGAGCTGTTCAAGGGCAAGAAGGGTGTGCTGTTTGGAGTTCCTGGGGCCTTCACCCCTGGAGCTTCCAAGACACACCTGCCAGGGTTTGTGGAGCAGGCTGAGGCTCTGAAGGCCAAGGGAGTCCAGGTGGTGGCCTGTCTGAGTGTTAATGATGCCTTTGTGACTGGCGAGTGGGGCCGAGCCCACAAGGCGGAAGGCAAGGTTCGGCTCCTGGCTGATCCCACTGGGGCCTTTGGGAAGGAGACAGACTTATTACTAGATGATTCGCTGGTGTCCATCTTTGGGAATCGACGTCTCAAGAGGTTCTCCATGGTGGTACAGGATGGCATAGTGAAGGCCCTGAATGTGGAACCAGATGGCACAGGCCTCACCTGCAGCCTGGCACCCAATATCATCTCACAGCTCTGAGGCCCTGGGCCAGATTACTTCCTCCACCCCTCCCTATCTCACCTGCCCAGCCCTGTGCTGGGGCCCTGCAATTGGAATGTTGGCCAGATTTCTGCAATAAACACTTGTGGTTTGCGGCCAGAATTC |
